# Tracheal Invasion and Cardiopulmonary Compromise From Primary Thyroid Lymphoma

**DOI:** 10.7759/cureus.19302

**Published:** 2021-11-06

**Authors:** Sean Fang, Vasileios Gkiousias, Lisi Hu, Karan Kapoor

**Affiliations:** 1 Department of Otorhinolaryngology, East Surrey Hospital, Redhill, GBR

**Keywords:** thyroid, cardiomyopathy, cardiopulmonary compromise, tracheal invasion, primary thyroid lymphoma

## Abstract

Rapidly expanding thyroid lesions with tracheal invasion are typical characteristics of anaplastic and undifferentiated thyroid carcinomas, but primary thyroid lymphoma (PTL) must also be considered as a differential. Aggressive thyroid lesions can compromise the airway through compression and/or direct invasion of the tracheal wall. We present a rare case of PTL in a 57-year-old female patient who presented with worsening orthopnoea and hoarseness, followed by shortness of breath, secondary to direct invasion and compression of the trachea resulting in pulmonary edema and cardiomyopathy, requiring intensive care input. In view of the extent of the disease and associated repercussions, the patient underwent total thyroidectomy and chemotherapy, as part of her therapeutic regime, with metabolic and cardiovascular remission achieved. Histological diagnosis confirmed diffuse large B-cell lymphoma (DLBCL). PTL is a rare condition, with few cases reported in the literature. Fine needle aspiration cytology (FNAC) used traditionally in the diagnosis of thyroid lesions is less informative in PTL and core needle and incisional biopsy techniques, coupled with CT, can provide diagnostic clarity. Due to the unusual nature of PTL, it can pose diagnostic and management difficulties. Further studies are required and a multi-professional tailored approach should be adopted for each patient until a therapeutic consensus can be reached.

## Introduction

Anaplastic and high-grade thyroid carcinomas typically present as rapidly expanding thyroid lesions with tracheal invasion. Nevertheless, primary thyroid lymphoma (PTL) must also be considered as a differential. PTL represents a rare entity characterized as lymphoma which only affects the thyroid gland itself or the thyroid gland and associated regional lymph node, without metastatic features at the time of diagnosis [1]. Differentiating between PTL and anaplastic thyroid carcinoma in patients with a rapidly expanding anterior neck mass can pose diagnostic challenges, with implications for subsequent management.

Tracheal invasion is a feature seen more commonly in anaplastic thyroid cancer due to the release of proteinases from neoplastic giant cells, which resemble osteoclasts associated with tracheal cartilage. Necrosis is uncommon in PTL and whilst extension beyond the thyroid capsule is relatively common, erosion of adjacent structures is unusual. Invasion of the trachea from PTL is very rare, especially prior to treatment, with only a few cases having been reported in the literature. 

Therapeutic approaches for PTL have also been laden with controversy, due to disease rarity and lack of prospective studies of large scale, with most cases requiring multi-professional input and management involving radiotherapy, chemotherapy, surgery, or a combination of those, on an individual case-by-case basis [1].

Here, we present a rare case of PTL that resulted in significant cardiopulmonary sequelae and required surgery, chemotherapy, and multidisciplinary team input for optimal management.

## Case presentation

A 57-year-old woman with known hypothyroidism presented to her general practitioner with a few months’ history of orthopnoea and intermittent hoarseness. An ultrasound showed right-sided thyroid enlargement with an isthmus nodule extending to the left lobe. She developed progressive tightness in the neck and was seen in the rapid access ENT clinic. Flexible nasendoscopy examination was normal and the patient underwent urgent ultrasound-guided fine needle aspiration cytology (FNAC), which suggested possible lymphocytic thyroiditis. Subsequent core biopsy showed scanty lymphocytes and was non-diagnostic.

Nineteen days following ENT review, she presented to the ED with worsening shortness of breath. The patient was diagnosed with type 2 respiratory failure with acidosis and raised troponin. A CT pulmonary angiogram (CTPA) on admission demonstrated severe ground glass shadowing. She was therefore admitted under the medical team with a working diagnosis of atypical pneumonia. An ECG demonstrated a reduced ejection fraction of 32% with left ventricle apical hypokinesia and lateral wall akinesia. Cardiac MRI demonstrated an apical left ventricle hypertrophy suggestive of cardiomyopathy. As a result, a diagnosis of Takotsubo stress cardiomyopathy was made.

During her inpatient stay, the patient suffered a cardiac arrest. Whilst spontaneous ventilation was achieved through cardiopulmonary resuscitation, worsening type 2 respiratory failure required intubation and mechanical ventilation in the ICU. The cause of arrest was attributed to acute respiratory distress syndrome secondary to acute pulmonary edema.

**Figure 1:**
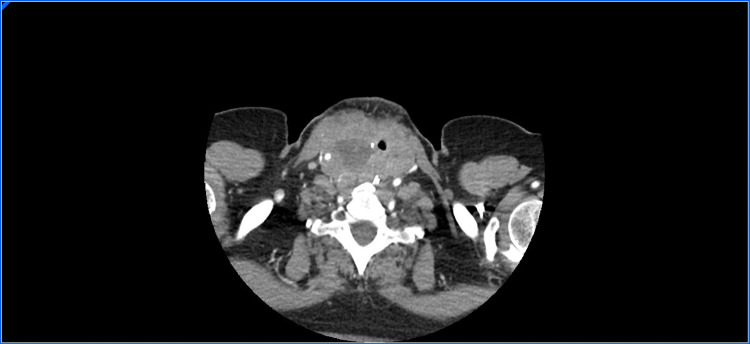
CT neck with contrast scan showing large thyroid lesion with significant tracheal compression, resulting in a minimal lumen size of 6 mm, without direct tracheal invasion.

In light of her cardiorespiratory deterioration, the admission CTPA was re-reviewed where a large goiter with heterogeneous enlargement was noted with significant tracheal compression resulting in a minimal lumen size of 6 mm (Figure 1).

No direct invasion of the trachea was seen on imaging. The radiological and clinical impression was that of a rapidly progressive and aggressive thyroid malignancy such as anaplastic carcinoma, rather than thyroiditis, as suggested on ultrasound. In view of the airway obstruction, the etiology of the ground glass appearance of the lungs was revised to pulmonary edema secondary to negative pressure respiration rather than an infective cause.

On day two of intubation, ENT was approached regarding surgical management of the airway, as the patient was deemed unsuitable for extubation due to the degree of tracheal compression. Therefore, a hemithyroidectomy of the larger right lobe of the thyroid was planned to decompress the trachea, gain a histological diagnosis and facilitate extubation.

Intraoperatively, the thyroid lesion was more consistent with an infiltrative pathology involving the strap muscles. The thyroid was grossly enlarged, homogeneously firm with an appearance more suggestive of lymphoma rather than carcinoma. The trachea was identified distally, and the thyroid cartilage was exposed to gain control of the field. The infiltration extended laterally toward the carotid sheath, hence an attempt at a formal hemithyroidectomy was not feasible. On dissecting the thyroid tissue from the trachea, it was evident on the right lateral aspect that there was a clear invasion into the lumen of the trachea. This was inspected using a 0-degree and 30-degree Hopkins rod. It was subsequently decided that a wedge resection of the thyroid down to the trachea and the formation of a tracheostomy would be the safest option. A size 8-0 cuffed Shiley tracheostomy tube was placed.

The patient was stepped down to a ward-based level of care after 17 days in ICU. Histological diagnosis revealed large pleomorphic lymphoid blasts, admixed with small lymphocytes and histiocytes. Immunohistochemistry revealed the lymphoid cells to be positive with CD20, PAX5, and BCL-2, with an expression of CD10 and BCL6. In addition, the cells were negative with CD3, CD5, CD21, CD23, cyclin D1 and MUM 1. Ki 67 proliferation fraction was found to be between 60 and 70%. Stains for TTF-1 and CK8/18 were negative. Consequently, the features were compatible with a high-grade B-cell lymphoma and most likely, diffuse large B-cell lymphoma (DLBCL).

**Figure 2:**
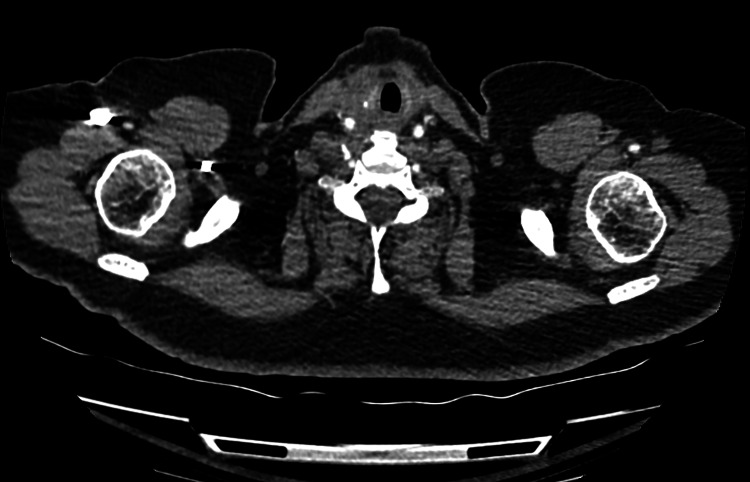
CT neck with contrast scan performed post total thyroidectomy and three cycles of R-CHOP chemotherapy, demonstrating marked regression of the previously visualized thyroid lesion and resolved airway compromise.

Under the care and close observation of the Haematology, Oncology, and Cardiology teams, the patient was commenced on six cycles of R-CHOP chemotherapy (four cycles at full dose, with two further cycles of rituximab). Her ejection fraction, troponin, and N-terminal brain natriuretic peptide (BNP) levels were closely monitored, as she was also given doxorubicin along her third cycle of chemotherapy. Marked improvement was noted on subsequent CT neck with contrast (Figure 2).

An end-of-treatment positron emission tomography-computed tomography (PET-CT) scan revealed complete regression of her DLBCL.

Since her discharge, the patient has been followed up closely by the Cardiology team and found to have restored ejection fraction on serial ECGs, as well as normal left ventricular size and function without any hypertrophy on follow-up cardiac MRI scan. The patient has also been decannulated successfully, following a brief period of observation in the hospital under the care of the ENT team.

The patient is currently being followed by the Hematology team at four-to-six monthly intervals and has been in remission for 18 months, without any palpable lymphadenopathy, organomegaly or masses, normalized weight, no systemic signs of infection, and normal blood test profiles (including thyroid function on maintenance dose levothyroxine).

## Discussion

Thyroid lymphoma can be classified into primary and secondary (non-thyroidal lymphoma, metastasizing to the thyroid gland). PTL is rare, accounting for less than 5% of all thyroid malignancies [2]. The thyroid gland does not typically contain lymphoid tissue therefore, PTL tends to occur in pathological glands. Autoimmune thyroiditis (Hashimoto’s thyroiditis) is the most common risk factor and is associated with 80% of cases of PTL and confers 40-80 times the risk of developing PTL compared to those without thyroiditis [3]. There is a female propensity for PTL (F:M ratio 3-4:1) [1], as was the case with the patient in this case report. Certain factors, such as age greater than 80 years, stage IIE-IV disease, no radiotherapy or surgery, and large B-cell or follicular histological profiles, tend to confer a worse prognosis [4]. Nevertheless, for early-stage PTL (IE and IIE) following appropriate management, a five-year survival rate of 100% has been reported for mucosa‐associated lymphoid tissue (MALT) and mixed-type (MALT, DLCBL) cases, and 87.5% for DLCBL cases, respectively [5]. 

Whilst traditional FNAC plays an important role in diagnosing thyroid nodules, it is of limited value in PTL due to the challenge in distinguishing between lymphoma, lymphocytic thyroiditis, and anaplastic thyroid carcinoma. However, advances in flow cytometry and immunohistochemistry have increased the sensitivity of FNAC in diagnosing PTL [6]. Core needle and incisional biopsy techniques provide significantly higher sensitivity and classify 95% of lymphomas in typical cases [7]. CT can suggest a diagnosis where anaplastic thyroid carcinoma displays more heterogeneous attenuation within the lesion, often with evidence of calcification and necrosis, whereas PTL and involved lymph nodes have a more homogenous appearance [4]. In our case, the diagnosis was reached following histology and immunohistochemistry after total thyroidectomy.

In terms of treatment for PTL, due to lack of large-scale studies and robust evidence, a bespoke, multidisciplinary approach has been advocated, with local control of disease achieved by surgery, radiotherapy, or a combination of both, and chemotherapy employed for hidden or widespread disease, in order for a more favorable long-term outcome to be achieved [1]. Combination treatment with chemotherapy and radiotherapy has been found to ensure a better outcome for five-year and ten-year survival (74% and 71%, respectively) when compared to monotherapy [5]. It is worth noting that no therapeutic guidelines exist for the management of PTL involving the airway, which has also seldom been reported in the literature [5].

In more detail, Van Ruiswyk J et al. reported two cases of PTL with airway obstruction, in a 49-year-old female and a 77-year-old male [8]. The former underwent subtotal thyroidectomy, followed by combination chemo-/radiotherapy, whereas the latter was managed with combination chemo-/radiotherapy alone, with the resolution of their symptoms and remission for up to two years later [8]. Melnyk A et al. reported a case of a 74-year-old female who was diagnosed with T-cell PTL, invading the trachea and causing extensive tracheal cartilaginous necrosis, and was managed with chemotherapy but unfortunately passed away shortly after [9]. Chen C et al. described the case of an 80-year-old female with a DLBCL with tracheal invasion, which was managed with chemotherapy and later tracheal dilation [4]. Finally, Kim EH et al. reported two cases of female patients (aged 73 and 77) diagnosed with DLBCL with tracheal and esophageal penetration, both treated with a combination of R-CHOP chemotherapy and radiotherapy, and both found to have complete regression of disease after four months [10].

The patient in this case report required a combination of surgery in the form of total thyroidectomy and adjuvant chemotherapy, due to the degree of disease invasion and severity of associated symptoms. Interestingly, when performed in patients affected by thyroid lymphoma, thyroidectomy has been found to be associated with a higher incidence of complications when compared to thyroidectomy performed for goiter or differentiated thyroid malignancy, due to increased difficulty of identifying key anatomical structures, such as the recurrent laryngeal nerves or parathyroid glands [11].

Relapse, either as local recurrence or distant metastatic disease, is likely after long-term primary management and can occur even after years [5], so follow-up at regular intervals, as has been the case thus far with our patient, is of paramount importance.

## Conclusions

PTL is a rare condition, associated with life-threatening complications. This case highlights the diagnostic challenge of differentiating between PTL and anaplastic thyroid carcinoma in patients with a rapidly expanding anterior neck mass, as there was progression to cardiopulmonary sequelae of airway compression resulting in negative pressure pulmonary edema and cardiomyopathy, necessitating intensive care support. Core needle and incisional biopsy techniques confer higher diagnostic yields than FNAC for diagnosing PTL, and CT can also aid in obtaining a diagnosis. A multidisciplinary team approach and coordination when dealing with complex thyroid cases are paramount. Lastly, in view of the uncommon nature of PTL, with few reported cases in the literature, and even fewer involving tracheal invasion, further studies are required to help guide management strategies and standardize guidelines in order for healthcare provisions to be optimized in affected patients.
